# Supplementary material for: MetaRibo-Seq measures translation in microbiomes
Source: Nat Commun. 2020 Jun 29;11:3268. doi: 10.1038/s41467-020-17081-z (PMC7324362; doi:10.1038/s41467-020-17081-z)
Supplement: Supplementary file 10 — Supplementary Data 7 [file 41467_2020_17081_MOESM10_ESM.zip › File2/Confidence_High_Taxonomy/393828_out.krona.html]

Javascript must be enabled to view this page.

members
magnitude
magnitudeUnassigned
count
unassigned
taxon
rank

393828\_out

3

3
superkingdom
2

1239
phylum
3

class
3
186801

order
3
186802

family
3
186803


SRS023914\_contig\_number\_18760
1898203
species
1

841
genus
2


SRS146812\_contig\_number\_contig-100\_1144.299869
2293144
1
species

1
species
301302

SRS144135\_contig\_number\_31157
